# Using camera traps to enhance community‐based management of subsistence hunting in the Amazon

**DOI:** 10.1111/cobi.70044

**Published:** 2025-05-30

**Authors:** Ricardo Sampaio, Ronaldo G. Morato, André Valle Nunes, Adriano G. Chiarello

**Affiliations:** ^1^ Centro Nacional de Pesquisa e Conservação de Mamíferos Carnívoros (CENAP) Instituto Chico Mendes de Conservação da Biodiversidade (ICMBio) Atibaia Brazil; ^2^ Pós Graduação em Biologia Comparada e Departamento de Biologia, Faculdade de Filosofia, Ciências e Letras de Ribeirão Preto (FFLCRP) Universidade de São Paulo (USP) Ribeirão Preto Brazil; ^3^ Panthera New York New York USA; ^4^ Knowledge Center for Biodiversity Belo Horizonte Brazil; ^5^ IPÊ – Instituto de Pesquisas Ecológicas Nazaré Paulista Brazil

**Keywords:** bottom‐up conservation strategies, MSOM, sustainable use reserves, tropical forests, wildlife management, bosques tropicales, estrategias de conservación ascendentes, gestión de fauna, MSOM, reservas de uso sostenible, 野生动植物管理, 自下而上的保护策略, MSOM, 可持续利用保护区, 热带森林

## Abstract

Community‐based management and monitoring of biodiversity has emerged as a cost‐effective strategy for providing credible data, informing decision‐making, and empowering local communities in resource governance and management. However, the establishment of community‐based management of subsistence hunting in the Brazilian Amazon has been hampered by legal uncertainty. Local regulations, such as the restriction or banning of mixed‐breed dogs in hunting, have been strengthened to address social conflicts and improve wildlife management, but the conservation effectiveness of such regulations has been questioned. We conducted a case study of community‐based decision‐making in a human community in the Riozinho da Liberdade Extractive Reserve in the southwestern Brazilian Amazon. This community established an informal agreement to limit the use of hunting dogs along one of the banks of the Liberdade River. After analyzing the results of 20 camera traps (CTs) placed in areas with and without the use of hunting dogs, the community strengthened their hunting agreement and decided to reinforce the agreement and ban this type of hunting completely. Subsequent to this decision, we analyzed the CT data and verified the negative effects of hunting with dogs on site‐level species richness, aggregate abundance and biomass, and the relative abundance and individual detection of some species. To strengthen community‐based subsistence hunting strategies in the Amazon and tropical forests in general, we suggest that camera trapping sampling of sites with different hunting management strategies and subsequent presentation to communities can facilitate local engagement, strengthen social and management rules, increase the decolonization of wildlife management, and ultimately expedite decision‐making processes to avoid the tragedy of the commons in similar tropical forest socioecological systems.

## Abstract (es)

Uso de cámaras trampa para mejorar la gestión comunitaria de la caza de subsistencia en la Amazonia

**Resumen:**

La gestión y el seguimiento comunitario de la biodiversidad ha emergido como una estrategia rentable para proporcionar datos fiables, fundamentar la toma de decisiones y capacitar a las comunidades locales en la gobernanza y la gestión de los recursos. Sin embargo, el establecimiento de la gestión comunitaria de la caza de subsistencia en la Amazonia brasileña se ha visto obstaculizado por la inseguridad jurídica. Se han reforzado las normativas locales, como la restricción o prohibición de perros mestizos en la caza, para abordar los conflictos sociales y mejorar la gestión de la fauna, aunque se ha cuestionado la eficacia de dichas normativas para la conservación. Realizamos un estudio de caso sobre la toma de decisiones comunitarias en una comunidad humana de la Reserva Extractiva de Riozinho da Liberdade, en el suroeste de la Amazonia brasileña. Esta comunidad estableció un acuerdo informal para limitar el uso de perros de caza a lo largo de una de las orillas del río Liberdade. Tras analizar los resultados de 20 cámaras trampa colocadas en zonas con y sin uso de perros de caza, la comunidad reforzó su acuerdo de caza y decidió reforzarlo y prohibir por completo este tipo de caza. Tras esta decisión, analizamos los datos de las cámaras trampa y comprobamos los efectos negativos de la caza con perros sobre la riqueza de especies a nivel de sitio, la abundancia y biomasa agregadas, y la abundancia relativa y detección individual de algunas especies. Para fortalecer las estrategias comunitarias de caza de subsistencia en la Amazonia y en los bosques tropicales en general, sugerimos que el muestreo con cámaras trampa de sitios con diferentes estrategias de gestión de la caza y su posterior presentación a las comunidades puede facilitar el compromiso local, fortalecer las normas sociales y de gestión, aumentar la descolonización de la gestión de la fauna y, en última instancia, agilizar los procesos de toma de decisiones para evitar la tragedia de los comunes en sistemas socioecológicos similares de bosques tropicales.

## 利用红外相机加强亚马逊地区基于社区的生计狩猎管理

**【摘要】** 基于社区的生物多样性管理和监测已成为一种具有成本效益的策略, 它能提供可靠的数据, 指导决策, 并增强当地社区在资源治理和管理方面的能力。然而, 在巴西亚马逊地区建立基于社区的生计狩猎管理仍一直面临法律不确定性的阻碍。为了解决社会冲突问题和改进野生动物管理, 政府加强了地方法规, 例如限制或禁止在狩猎中使用混种犬, 但人们对这些法规的保护效果仍存在质疑。我们在巴西亚马逊地区西南部的Riozinho da Liberdade采掘保护区的一个社区开展了一项基于社区决策的案例研究。该社区达成了一项非正式协议, 限制在Liberdade河的一个河岸用猎犬来狩猎。在分析了使用及不使用猎犬区域放置的20个红外相机的结果后, 该社区加强了他们的狩猎协议, 并决定完全禁止这种狩猎方式。在做出这一决定后, 我们对红外相机数据进行了分析, 验证了使用猎犬狩猎对位点水平的物种丰富度、总丰度和生物量, 以及一些物种的相对丰度和个体出现情况存在负面影响。为了加强亚马逊和整个热带森林中基于社区的生计狩猎管理策略, 我们建议对采用不同狩猎管理策略的地点进行红外相机监测, 并向社区展示监测结果, 从而促进当地社区的参与, 加强社会和管理规则, 提高野生动物管理的去殖民化程度, 最终加快决策进程, 避免类似的热带森林社会生态系统中的公地悲剧。**【翻译:胡怡思;审校:聂永刚】**

## INTRODUCTION

Community‐based management and monitoring of biodiversity have increased in the last 20 years and occur on virtually all inhabited continents (Danielsen et al., 2021). It is considered a cost‐effective strategy that can provide credible data, inform decision‐making, and empower local communities to govern local resources (Campos‐Silva & Peres, 2016; Danielsen et al., 2021; dos Reis & Benchimol, 2023; Lopes et al., 2021).

The establishment of community‐based management programs of subsistence hunting in Brazilian Amazon, however, is compromised due to legal uncertainties (Antunes et al., 2019; Bragagnolo et al., 2019). Hunting is prohibited in Brazil (Law 5,197/67), but it is allowed for Indigenous populations (Law 6001/1973) and may be legal for subsistence of non‐Indigenous local human populations (Laws 9,605/98 and 10,826/2003), although there is no specific legislation defining and regulating subsistence hunting in Brazil. Consequently, most initiatives are based on existing local rules of hunting and wildlife management, where local people collectively negotiate and legitimate their local practices and cultural norms in a decision‐making process (Vieira et al., 2019). In general, management plans of sustainable use reserves (SURs) in the Brazilian Amazon incorporate local rules of wildlife management as official rules (Vieira et al., 2019). This is the case in some SURs that prohibit the use of dogs in hunting.

The use of domestic dogs increases the efficiency of hunting and consequently provides more wild meat for local people (Constantino, 2019; Koster & Noss, 2013; Santos et al., 2022). Hunting with dogs tends to target the fast‐breeding and persistently hunted species (Constantino, 2019; Koster & Noss, 2013; Redford & Robinson, 1987). This pattern could be associated with the sustainability of subsistence hunting because hunting with dogs may reduce local hunting pressure on slow‐reproducing species, which are the most sensitive to hunting (Constantino, 2019; Koster, 2008; Koster & Noss, 2013). In addition, this hunting strategy is typically restricted to areas close to communities, especially near agricultural fields (Constantino, 2019; Koster, 2008).

However, dogs do not respect the boundaries of hunting areas, and not everyone can afford to buy them (de Almeida & Pantoja, 2005). Consequently, restrictions on the use of domestic dogs in hunting activities have emerged in the rural Brazilian Amazon as a measure to reduce potential social conflicts in local communities (Dias & de Almeida, 2004). This local rule may have been created and imposed by the rubber patrons during the rubber boom in the Amazon and subsequently followed by rubber tappers and Indigenous people (Constantino, 2019; de Almeida & Pantoja, 2005). However, even among local people in the Brazilian Amazon, the effectiveness of banning hunting with dogs is questionable. Not all local hunters agree with and respect these rules, and some believe that hunting with dogs is no more harmful to wildlife than hunting without dogs (R.S., personal observation), and they continue to use dogs because the protein yield is higher (Constantino, 2019; Koster, 2008; Koster & Noss, 2013).

We conducted a case study of community‐based decision‐making related to the regulation of hunting with dogs. In 2016, the community restricted hunting with dogs on one bank of the Liberdade River in 2016. In 2018, we deployed camera traps (CTs) on both banks of the river and showed the photographs from these cameras to the community. We hoped to provide insights into how CT data used in a photographic presentation can facilitate local engagement; strengthen, prioritize, and legitimize social and management rules; and expedite decision‐making processes for multiple use of resources in tropical SURs. Local people's involvement can contribute to the decolonization of tropical wildlife management (Domínguez & Luoma, 2020; van Vliet, 2018).

## METHODS

### Study area

**FIGURE 1:**
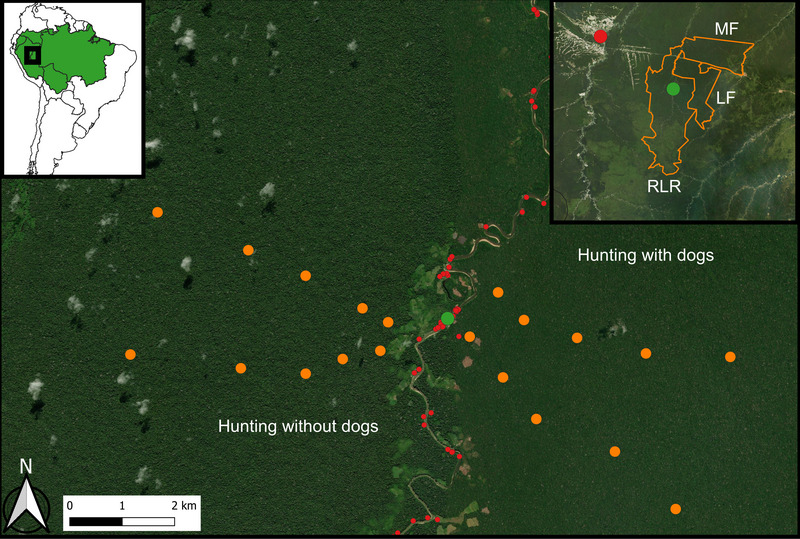
Location of the Periquito community studied (green dot) in the Riozinho da Liberdade Extractive Reserve (RLR); Liberdade (LF) and Mogno (MF) State Forests (orange polygons); 20 deployed camera traps (orange dots); households (red dots); and areas where hunting with dogs is allowed and not allowed (green in upper left panel, Amazon lowlands).

The Riozinho da Liberdade Extractive Reserve (LER) is an SUR of 340,000 ha in southwestern Amazon in the Liberdade River sub‐basin of the Juruá River in Cruzeiro do Sul, Acre state, Brazil. The vegetation consists of ombrophilous forest with an open canopy and widespread occurrence of palm trees and bamboos (*Guadua* spp.) (Acre, 2006). The reserve is inhabited by *ribeirinhos*, former rubber tappers who live in semisubsistence communities of mixed descent. Approximately 1200 people live in 21 villages in the reserve. Hunting and cassava (*Manihot esculenta*) cultivation are widespread (Nunes et al., 2019). The community we studied is called Periquito (Figure 1) and contained approximately 25 households. In 2016, perceiving a reduction in game stock in the surrounding forest, they established a local hunting agreement that prohibited the use of mixed‐breed dogs in hunting on the left bank of Liberdade River; dogs were allowed on the right bank. The community agreed to annually discuss and evaluate the effectiveness of their agreement and to decide whether to continue it.

### CT sampling and studied species

In 2018, R.S. set up 20 CTs after consulting with and obtaining permission from the community of Periquito and following authorization from the Instituto Chico Mendes de Conservação da Biodiversidade (ICMBio) (SISBIO 59835‐3). The CTs were remotely activated, digital, and triggered by motion or temperature change (Bushnell Trophy‐Cam) in the primary terra firme forests surrounding the community on both banks of the Liberdade River. Ten CTs were deployed on each bank of this river at varying Euclidean distances from the community, ranging from 0.54 to 5.1 km (mean = 2.7 km [SD 1.4]). The CTs were installed approximately 30 cm above the ground and at least 20 m away from the nearest human trail. Cameras operated continuously for 49 days without bait. We focused on 29 terrestrial and semiterrestrial vertebrate species (or species functional groups) that could reliably trigger the CT sensor and be identified to at least genus level by visual inspection of the photographs. These included birds and mammals with body weights >400 and 700 g, respectively (Table 1).

**TABLE 1:** List of the 29 focal species examined in a study of hunting with dogs conducted in Periquito community (RESEX Riozinho da Liberdade).

Scientific name	English name	Code	Game	Size (kg)^*^	Recorded by camera trap
Mammals					
*Tapirus terrestris*	Lowland tapir	Tapi.te	Yes	160	Yes
*Panthera onca*	Jaguar	Pant.on	Yes	80	No
*Puma concolor*	Puma	Puma.co	Yes	45	No
*Tayassu pecari*	White lipped peccary	Taya.pe	Yes	32	No
*Mazama americana*	Red brocket deer	Maza.am	Yes	30	Yes
*Priodontes maximus*	Giant armadillo	Prio.ma	Yes	30	No
*Dicotyles tajacu*	Collared peccary	Dico.ta	Yes	25	Yes
*Mazama nemorivaga*	Gray brocket deer	Maza.ne	Yes	18	Yes
*Leopardus pardalis*	Ocelot	Leop.pa	Yes	15	Yes
*Cuniculus paca*	Paca	Cuni.pa	Yes	9.5	Yes
*Puma yagouaroundi*	Jaguarundi	Puma.ja	Yes	8	No
*Leopardus wiedii*	Margay	Leop.wi	Yes	6	Yes
*Nasua nasua*	South American coati	Nasu.na	Yes	5.1	Yes
*Dasyprocta fuliginosa*	Agouti	Dasy.fu	Yes	4.5	Yes
Nonspecific small Cingulata	Armadillos	Dasypus	Yes	6	Yes
*Hadrosciurus spadiceus*	Southern Amazon red squirrel	Hadro.sp	Yes	1.2	Yes
*Myrmecophaga tridactyla*	Giant anteater	Myrm.tr	No	30.5	Yes
*Atelocynus microtis*	Short‐eared dog	Atel.mi	No	7.75	Yes
*Speothos venaticus*	Bush dog	Speo.ve	No	6	No
*Procyon cancrivorus*	Crab‐eating raccoon	Proc.ca	No	5.4	No
*Eira barbara*	Tayra	Eira.ba	No	4.85	Yes
*Tamandua tetradactyla*	Southern tamandua	Tama.te	No	4.5	Yes
*Didelphis marsupialis*	Common opossum	Dide.ma	No	1.09	Yes
*Myoprocta pratti*	Acouchi	Myop.pr	No	0.75	Yes
Birds					
*Mitu tuberosum*	Curassow	Mitu.tu	Yes	3	No
*Penelope jacquacu*	Spix's guan	Pene.ja	Yes	1.2	Yes
*Psophia leucoptera*	Trumpeters	Psop.le	Yes	1.28	Yes
*Tinamus* spp.	Large tinamou	Tina.sp	Yes	1.2	Yes
*Crypturellus* spp.	Small tinamou	Cryp.sp	Yes	0.42	Yes

^a^ Abrahams et al. (2017).

### Photography presentation

After the removal of the CTs from the forests, the community members asked R.S. to show the photos taken by the CTs to the whole community in a meeting on an image projector. Virtually all community members attended this presentation, and the only intervention by R.S. during the meeting was to exhibit the images and indicate where the camera was that took the picture (river's bank and the distance from the community). The exposure time of each photo varied during the presentation. When the audience identified an animal in the photos, some comments were made by them, and R.S. displayed the next photo only after the audience finished commenting. When the audience did not comment about the animals appearing in the photos or when there was no animal in the photo, R.S. moved on to the next photo and so on until the last photo from the cameras had been shown.

### Statistical analyses

All statistical analyses were performed using R 4.0.4. (R Core Team, 2021). We rescaled Euclidean distances from the Periquito community to CT (mean = 0 and SD = 1) to improve model convergence and facilitate comparisons of variable effect sizes (Harrison et al., 2018). We assumed that our sampling design and temporal replicates met key model assumptions of multispecies occupancy models (MSOMs), such as demographic closure and accurate species identification (Devarajan et al., 2020). To prevent possible temporal autocorrelation in species detection (Goldstein et al., 2024), we created detection histories per CT based on detection and nondetection records over 5‐day intervals for all species (detectionHistory function in camtrapR package [Niedballa et al., 2016]) and fitted 2 single‐season MSOMs following the methods of Yamaura et al. (2011).

The MSOM framework is based on the assumptions that individuals of species *i* at site *j* are independently detected with probability *r_ij_* and that the probability of detection for species *i* at site *j* depends on the local abundance of species *i*. The probability of detection for species *i* at site *j* depends on the local abundance of species *i*:

(1) $$p_{ij}=1-{\left(1-r_{ij}\right)}^{Z_{ij}},$$

where *p_ij_* is the detection probability of species *i* at site *j* and *Z_ij_* is the abundance of species *i* at site *j*. The species detection frequency *Y_ij_* over *V_j_* visits follows a binomial distribution with parameter *p_ij_* (*Y_ij_* ∼ binomial [*V_j_*, *p_ij_*]), whereas the local population size *Z_ij_* follows a Poisson distribution with mean λ*_ij_* [*Z_ij_* ∼ Poisson (λ*_ij_*)]. Both *r_ij_* and λ*_ij_* parameters can be modeled with explanatory variables. The estimates of variable effects for all species can be improved in MSOMs (Zipkin et al., 2010) because the parameters of interest for each species share a common distribution governed by hyperparameters of the community (Kéry & Royle, 2015).

Due to our small sample size, we devised a strategy to minimize the number of parameters being estimated by the models. To do this, we ran 2 models, including the predictor effects in only one of our parameters of interest (https://github.com/rsampaio‐cenap/Sampaio_et_al_hunting_agreement_SM). First, we examined the effects of the community distance, hunting strategy, and the interaction of these predictors on relative abundance (Model 1); individual detection was kept constant. Second (Model 2), we investigated the effect of the predictors in Model 1 on individual detection probability, with abundance kept constant.

In our 2 models, the local abundance parameters *Z_ij_* represented the number of individuals available for detection around each CT (Royle & Nichols, 2003), and they can be interpreted as a relative measure of the intensity of species habitat use (Martijn et al., 2023; Nakashima, 2020), especially for larger‐bodied species (>15 kg), where an individual may be detected by more than one CT. In all models, we used a data augmentation procedure that added all‐zero detection histories for 10 potentially undetected species to estimate species richness at the site level (Dorazio et al., 2006).

In Model 1, in which independent variables affected relative abundance, we computed the posteriors of species richness per site, aggregated abundance of all species per site (hereafter, abundance), and aggregated biomass per site (hereafter, biomass) (calculated as the sum of species’ relative abundance times their mean body mass). We fitted 3 Bayesian linear mixed models (LMMs) to assess the effects of community distance and hunting strategy on the posterior estimated values of species richness, aggregated abundance, and biomass; site name was included as a random variable.

We fitted the 2 MSOMs with the rjags package (Plummer, 2022) and the 3 LMMs with the brm function of the brms package (Bürkner, 2017) and used Markov chain Monte Carlo (MCMC). We used noninformative priors for all parameters, 3 MCMCs (Gelman & Shirley, 2011), and 100,000 iterations per chain with a burn‐in of 50,000 and a thinning rate of 100. We evaluated chain convergence with the Gelman–Rubin convergence diagnostic (Rhat ≤ 1.1 [Brooks & Gelman, 1998]) for each parameter of interest ( https://github.com/rsampaio‐cenap/Sampaio_et_al_hunting_agreement_SM) and visually inspected trace plots to confirm convergence. We considered there was evidence of support for the effect of an explanatory variable when the estimated 95% posterior credible interval did not include zero.

## RESULTS

### Photograph presentation

After showing the entire Periquito community the CT photos, a spontaneous, very rich discussion about the biology of the game species and the effectiveness of their hunting agreement started. R.S. perceived that the community agreed that more game photos were seen in areas where hunting was done without dogs. Of the 5501 photographs taken by the 20 CTs in 2018, 3705 included focal species records, of which 2025 were made on sites without hunting with dogs and 1680 on sites where hunting with dogs occurred.

In 2019, R.S. received a letter containing the official minutes of a meeting in Periquito (https://github.com/rsampaio‐cenap/Sampaio_et_al_hunting_agreement_SM). The letter stated that the 2018 presentation of the CT photos reinforced the effectiveness of their hunting agreement strategy; they decided to continue restricting the use of hunting with dogs; hunters agreed to stop breeding of hunting dogs; and, after 1 year, they would stop hunting with dogs on both sides of the river.

### Posterior data analyses

**FIGURE 2:**
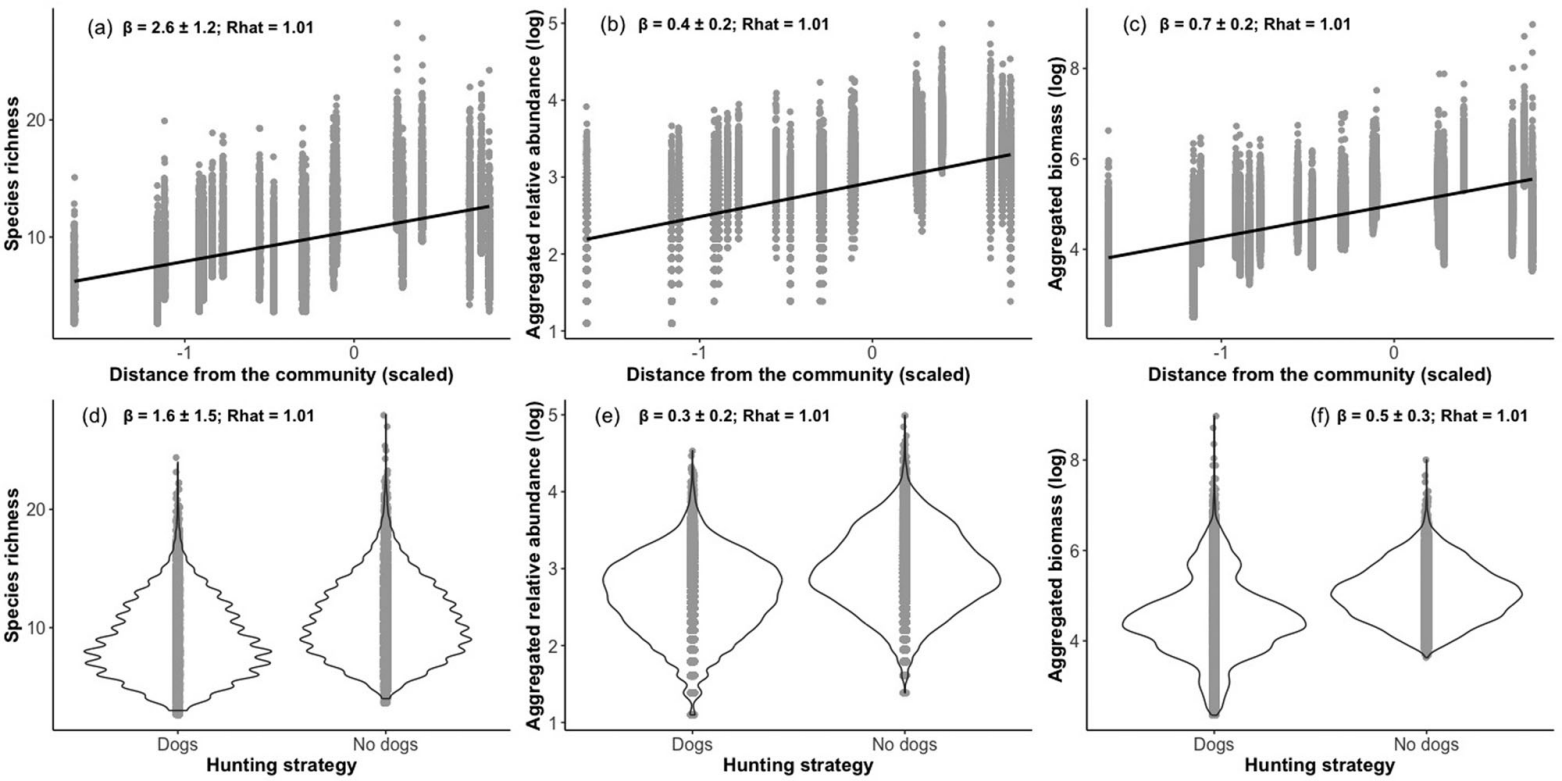
Relationship from Bayesian linear mixed models between (a) estimated species richness, (b) relative abundance, and (b) biomass and scaled values of the distance from community and between the variations in the (d) estimated species richness, (e) relative abundance, and (f) biomass relative to hunting strategy (dogs, right river bank, hunting assisted by domestic dogs allowed; no dogs, left river bank, hunting with dogs not allowed; gray dots, estimated values of the dependent variables). Shown are posterior mean values of the variable coefficient (β), confidence intervals, and the Gelman–Rubin convergence model value (Rhat).

The 20 CTs recorded 21 native species (17 mammals and 4 birds) (Table 1). Our post hoc analyses of CT data showed that site‐level species richness, aggregated relative abundance, and biomass were lower near Periquito and in areas where hunting with dogs was allowed (Figure 2).

**FIGURE 3:**
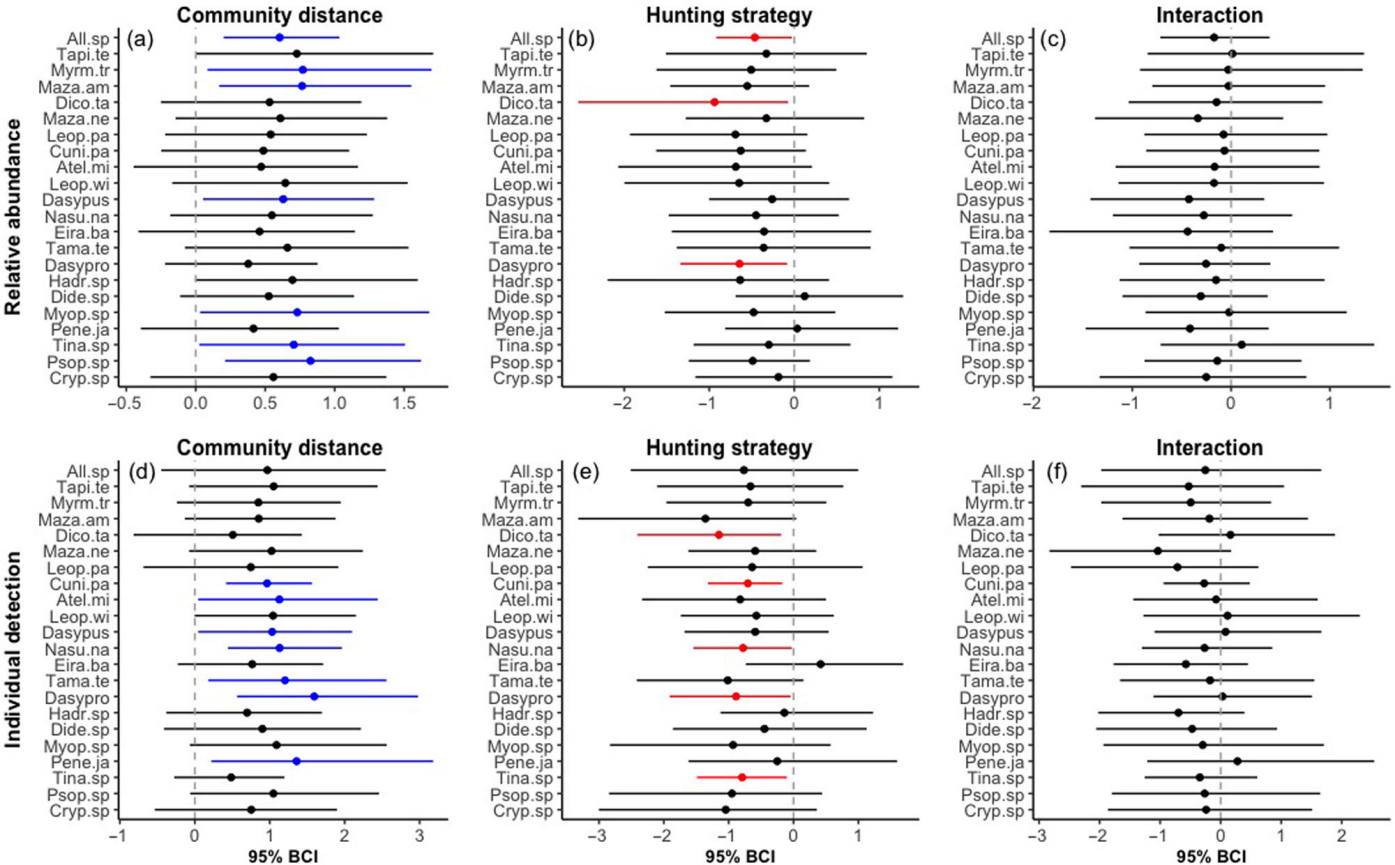
Magnitude and direction effects (Bayesian credible interval [BCI]) for the posterior distribution of the predictor effects of (a, d) distance from community, (b, e) hunting strategy (without dogs as reference parameter), and (c, f) the interaction of distance and hunting strategy on the relative abundance of species detected with camera traps and on the individual detection probability of all species (All.sp) (species community's hyperparameter) and of individual species (dots, mean of estimated values of dependent variables; blue, positive effects; red, negative effects; black, no significant effects). Species are ordered according to their mean body mass.

The values of the relative abundance of all species (All.sp; species community's hyperparameter), tapir (*Tapirus terrestris*) (marginal effect), giant anteater (*Myrmecophaga tridactyla*), red brocket deer (*Mazama americana*), small cingulata (*Dasypus*), acouchi (*Myoprocta pratti*), large tinamous (*Tinamus* spp.), and trumpeter (*Psophia leucoptera*) and the individual detections of tapir (*Tapirus terrestris*, marginal effect), red brocket deer (marginal effect), gray brocket deer (*Mazama nemorivaga*, marginal effect), paca (*Cuniculus paca*), short‐eared dog (*Atelocynus microtis*), margay (*Leopardus wiedii*, marginal effect), small cingulata, coati (*Nasua nasua*), southern tamandua (*Tamandua tetradactyla*), agouti (*Dasyprocta fuliginosa*), acouchi (marginal effect), Spix's guan (*Penelope jacquacu*), and trumpeter (marginal effect) increased as the distance between the CT and Periquito increased (Figure 3a,d). Abundance of all species, collared peccary (*Dicotyles tajacu*) and agouti and the individual detection probability of red brocket deer (marginal effect), collared peccary, paca, coati, southern tamandua (marginal effect), agouti, and large tinamou were lower in areas where hunting with dogs was allowed than in areas where it was not allowed (Figure 3b,e).

## DISCUSSION

Our case study showed how evidence‐based information can support and strengthen local strategies for subsistence hunting in tropical forests, when this evidence is presented to local communities in an after‐the‐fact format, such as the informal presentation of CT photographs, and through subsequent statistical analyses. According to the minutes of the 2019 Periquito community meeting, the hunting agreement strategy was strengthened and local hunters banned hunting with dogs. This agreement created and executed by the local population emphasizes the importance of governance and cooperation to sustain natural resources (Ostrom, 1990). By integrating these principles, the agreement may be inclusive, thus promoting the sustainable use of resources. This holistic approach ensures that the local community can manage the complexities of tropical forest management.

We identified the negative effects of hunting with dogs on species richness, aggregated abundance and biomass, and relative abundance and individual species detection of some game species. Our study provides valuable insights into how community‐based hunting management strategies can improve resources for local communities and biodiversity conservation, especially in areas where the understory hunting pressure may have depleted species sensitive to hunting.

Our study location appears to have depleted numbers of large forest vertebrates. For example, our CTs did not record the white‐lipped peccary (*Tayassu pecari*) or curassow (*Mitu tuberosum*), 2 species that are sensitive to hunting pressure near human communities (Sampaio et al., 2023), although white‐lipped peccary populations fluctuate naturally (Fragoso et al., 2022). Local residents reported that these species were not present in Periquito (R.S., unpublished data).

### Community distance effects

The proximity of CTs to Periquito was important in determining wildlife declines. At the CT sites close to Periquito, species richness, abundance, and biomass were lower than at other CT sites. The abundance of some species and individual species detection of both game (tapir, 2 brocket deer, paca, small cingulata, agouti, large tinamous, and trumpeter) and nongame species (giant anteater, short‐eared dog, margay, southern tamandua, and acouchi) were also affected by proximity to Periquito. This halo of impacts near human settlements is consistent with several large‐scale studies in tropical forests (Abrahams et al., 2017; Beirne et al., 2019; Koerner et al., 2017; Sampaio et al., 2023; Van Kuijk et al., 2022).

Based on central‐place foraging behavior of tropical forest subsistence hunters (Sirén et al., 2004), which results in a higher hunting effort closer to households (Griffiths et al., 2022), we believe that the reduced wildlife abundance near Periquito was directly related to the removal of individuals through hunting. This result may be related to agonistic encounters with humans or dogs near the village, which may alter the habitat use of wildlife species. However, this result may also be influenced by other types of subcanopy forest degradation (such as logging and fire incidence) that likely occurred near Periquito.

### Effects of hunting with dogs

We cannot rule out the possibility that some confirmation bias may have influenced the community's perceptions during the photograph presentation. The presentation of over 5000 photos may have been tiring and disorganized, causing confusion as to which side of the river a particular photo belonged to. However, there was a motivated and heated discussion about the hunting agreement, and we observed a 20% increase in the number of photos of the focal species in the sites where hunting with dogs did not occur.

We caution that the generalization of our results on the effect of hunting with dogs is limited due to our sampling design, which included only one human community and a small number of CTs (20). In addition, there may be environmental variables that altered the game species’ carrying capacity on different banks of the Liberdade River, such as the differing vertical distances to the nearest drainage (Rennó et al., 2008) near our CTs (https://github.com/rsampaio‐cenap/Sampaio_et_al_hunting_agreement_SM), which may also influence the patterns of abundance and individual detection of species derived from CT data. Furthermore, we did not have data to investigate potential differences in hunting pressure between the 2 riverbanks (i.e., whether the frequency of hunting events was higher where hunting with dogs took place after the agreement was enacted). These potentially confounding factors could have masked the effects of the hunting strategy.

Despite the limitations of our sampling design, we found negative impacts of hunting with dogs on wildlife in Periquito. These impacts were perceived by local people in the 2018 photo presentation and captured in our post hoc analyses. Reductions in species richness, aggregated abundance and biomass, abundance, and individual detection of 4 fast‐breeding species less sensitive to hunting (collared peccary, paca, agouti, and large tinamous), as well as red brocket deer and coati, occurred where hunting with dogs was allowed.

Hunting with dogs in other communities of the extractive reserve is mainly focused on acouchis (26%), followed by pacas (18%) and collared peccaries (11%) (A.V.N., unpublished data). Hunting dogs forage in the forest, usually emitting rapid barks. These dogs frequently chase their prey to corner them in holes, tree hollows, or some specific location. When this happens, the dogs emit a distinctive bark to indicate that it has cornered an animal (R.S., personal observation). We reason that this strategy could particularly affect species that seek shelter in tree hollows and holes in the ground when fleeing, such as the collared peccary, coati, agouti, and paca (Beisiegel, 2006; Figueroa‐de‐León et al., 2016; Smith, 1976; Smythe, 1978), although coatis also escape by climbing trees or freezing (R.S., personal observation).

Our results indicated that community‐based strategies focused on banning or reducing this practice may be beneficial for local people and biodiversity. It is reasonable to assume that the reduction in wildlife populations was due to the greater efficiency of hunting with dogs (Constantino, 2019; Koster & Noss, 2013; Santos et al., 2022). Hunting dogs may exhibit unselective foraging behavior that frightens nontarget individuals (da Cunha & Almeira, 2000; Koster, 2008). In addition, the signs of dogs, such as urine, feces, and barking, which can carry over long distances in the forest, can also affect wildlife behavior (dos Santos et al., 2018). This could reduce the habitat use of the species in areas where hunting with dogs occurs, thus reducing the number of individuals available for detection in CTs, which was perceived by local people and in our posteriori analyses.

However, this reasoning is at odds with some studies that have failed to provide robust evidence that hunting with dogs has a greater impact on game populations than hunting without dogs (Constantino, 2019; Koster & Noss, 2013; Santos et al., 2022). These authors postulated that hunting with dogs targets the fast‐breeding species mentioned above. Our results suggest that this hunting strategy affects not only their local abundance but also their individual detection. Another aspect that may be related to these contrasting results is the fact that our study was conducted where species have been somewhat depleted and in a non‐Indigenous population, where the hunting pressure from hunting with dogs may be greater than in other areas.

We postulated several factors that make it difficult to generalize the effects of hunting with dogs on game species, and further studies at large spatial scales in tropical forests would be welcome. However, our study case showed a correlation between the negative effect perceived by the local population and the results of the post hoc analysis of CT data, where the presentation of CT photos helped the Periquito community make quick decisions about their wildlife management. This experience may be relevant under similar conditions in other tropical forest socioecological systems.

### Conservation considerations

Our study provided relevant insights for strengthening community‐based strategies for subsistence hunting in the Amazon, where local rules need to be constantly reviewed and strengthened (Vieira et al., 2019). The strengthening of hunting agreements in Periquito highlights the importance of mutual cooperation and shared responsibility among community members in the management of common resources (Sabourin, 2023). This principle can promote a sense of ownership and collective action in management rules. The Periquito community's decision to apply the prohibition of hunting with dogs to both sides of the river became an official rule for LER (Brasil, 2020). Using strategies similar to these, stakeholders can achieve the same potential benefits in other socioecological systems in tropical forests around the world.

Bottom‐up strategies of biodiversity management have had good results in several tropical socioecological systems (Danielsen et al., 2021; Londres et al., 2023). However, the lack of involvement of local people is the main obstacle to the effectiveness of local game management in tropical forests (dos Reis & Benchimol, 2023). We found that CTs can provide immediate photographic evidence to local people; that the photographic evidence can be well received by a community; and that photographs can be sufficient as a basis for changing local wildlife management strategies. This sampling strategy engages, strengthens, prioritizes, and legitimizes local stakeholders and contributes to the decolonization of wildlife management in the tropics because local, traditional, and Indigenous people are frequently excluded in the tropical wildlife management agenda (Domínguez & Luoma, 2020; van Vliet, 2018).

Our case study can be replicated in communities that are in need of immediate hunting management strategies or other natural resource management strategies in tropical forests in general. In these areas, the effectiveness of local agreements can be easily measured, validated, and deliberated by local people, and these steps increase communities’ governance and legitimacy. CTs are inexpensive and can be operated by local people, and data from CTs can be used immediately to promote local commitment to community‐based strategies for local resource use.
